# Handheld Computer Devices to Support Clinical Decision-making in Acute Nursing Practice: Systematic Scoping Review

**DOI:** 10.2196/39987

**Published:** 2023-02-13

**Authors:** David Glanville, Anastasia Hutchinson, Damien Khaw

**Affiliations:** 1 eLearning Department Academic & Medical Division Epworth HealthCare Richmond Australia; 2 Centre for Quality and Patient Safety Research – Epworth HealthCare Partnership Institute of Health Transformation Deakin University Burwood Australia

**Keywords:** handheld computer devices, smartphones, mobile computing, mobile health, nursing, acute care, decision-making, clinical decision-making, scoping review, mobile phone

## Abstract

**Background:**

Nursing care is increasingly supported by computerized information systems and decision support aids. Since the advent of handheld computer devices (HCDs), there has been limited exploration of their use in nursing practice.

**Objective:**

The study aimed to understand the professional and clinical impacts of the use of mobile health apps in nursing to assist clinical decision-making in acute care settings. The study also aimed to explore the scope of published research and identify key nomenclature with respect to research in this emerging field within nursing practice.

**Methods:**

This scoping review involved a tripartite search of electronic databases (CINAHL, Embase, MEDLINE, and Google Scholar) using preliminary, broad, and comprehensive search terms. The included studies were hand searched for additional citations. Two researchers independently screened the studies for inclusion and appraised quality using structured critical appraisal tools.

**Results:**

Of the 2309 unique studies screened, 28 (1.21%) were included in the final analyses: randomized controlled trials (n=3, 11%) and quasi-experimental (n=9, 32%), observational (n=10, 36%), mixed methods (n=2, 7%), qualitative descriptive (n=2, 7%), and diagnostic accuracy (n=2, 7%) studies. Studies investigated the impact of HCDs on nursing decisions (n=12, 43%); the effectiveness, safety, and quality of care (n=9, 32%); and HCD usability, uptake, and acceptance (n=14, 50%) and were judged to contain moderate-to-high risk of bias. The terminology used to describe HCDs was heterogenous across studies, comprising 24 unique descriptors and 17 individual concepts that reflected 3 discrete technology platforms (“PDA technology,” “Smartphone/tablet technology,” and “Health care–specific technology”). Study findings varied, as did the range of decision-making modalities targeted by HCD interventions. Interventions varied according to the level of clinician versus algorithmic judgment: unstructured clinical judgment, structured clinical judgment, and computerized algorithmic judgment.

**Conclusions:**

The extant literature is varied but suggests that HCDs can be used effectively to support aspects of acute nursing care. However, there is a dearth of high-level evidence regarding this phenomenon and studies exploring the degree to which HCD implementation may affect acute nursing care delivery workflow. Additional targeted research using rigorous experimental designs is needed in this emerging field to determine the true potential of HCDs in optimizing acute nursing care.

## Introduction

### Background

The commercial release of smartphones in 2007 initiated a revolution in handheld device ownership, facilitating multimodal instant communication options and the rapid evolution of mobile health apps that provide instantaneous access to web-based information and resources [1]. Handheld computer devices (HCDs) with internet connected functionality are now widely used to support health practitioner communication, documentation, education, research, and clinical decision-making across health care systems, including acute practice settings. The use of HCDs may offer advantages over fixed bedside information systems through their portability, rapid deployability [2], and cross-platform integration across care settings [3]. However, to effectively promote the quality and safety of care, the rapidly evolving landscape of HCDs in clinical practice requires a strong evidence-based foundation [4,5]. However, presently, the development of HCD-based apps and their use at the point of care have outpaced their empirical testing such that their overall effect on patient outcomes remains unclear [4,6,7].

HCDs provide nursing staff with a powerful and accessible mobile platform for a range of decision support applications. Tiffen et al [8] defined nurses’ clinical decision-making as, “the process of choosing between alternatives or options through the gathering and evaluation of data, from which a decision, judgement or intervention is formulated.” HCDs may support best nurse decision-making at the point of care through the provision of evidence-based prompts and the use of mobile computing to quantify key clinical markers or produce an algorithmic judgment from a combination of available information [9]. Despite sharing common principles of evidence-based decision-making with other health disciplines [10], nursing-specific knowledge and the training and scope of practice render the processes of nurse decision-making distinctive [11,12]. However, there are strong theoretical and empirical reasons to expect improvements to nursing care if structured decision aids can be carefully integrated into nursing practice. Since the 1950s, evidence from the psychological sciences has demonstrated that the incorporation of evidence-based, algorithmic judgments typically outperforms unaided clinical judgments across a wide range of both medical and nonmedical applications [13-15]. The use of HCDs in the clinical space reflects the application of current mobile technology to facilitate such judgments at the point of care.

### Prior Work

Reviews of the extant literature have typically explored HCD use by nonnurses [16,17], did not differentiate between professions [18,19], or have explored nurses’ use of information and communication technology broadly, without specifically focusing on the bedside use of HCDs in acute settings [20-23]. However, in 2014, Mickan et al [9] reported the findings of a systematic review on the use of HCDs to support information access and clinical decision-making at the point of care. The authors noted that at that time, 7 randomized controlled trials had explored this concept, finding that the use of handheld tools improved knowledge acquisition and safety with respect to point-of-care decision-making. However, all the identified studies were based on earlier generation PDA technology and primarily evaluated physicians’ (5/7, 71%) rather than nurses’ use. It has been argued that the nursing profession lags other health care professions in its acceptance of using such technologies. However, it is to be noted that nurses in some health care settings have been prohibited from using digital tools independently to support clinical decision-making and practice delivery [24,25]. Therefore, although this earlier review was conducted after the smartphone era, a new systematic review of the literature is required to capture the impact of recent technological innovations.

In this paper, we report the findings of a scoping review that sought to identify and evaluate the body of published empirical literature investigating the use and effectiveness of HCDs in supporting nurses’ clinical decision-making in the acute health care settings. This review aimed to summarize the extent, quality, characteristics, and scope of published research and identify key nomenclature with respect to this emerging field.

## Methods

### Design

To address the above aims, we undertook a scoping review involving both systematic electronic database searches and hand searches of the reference lists of the included studies. The PRISMA-ScR (Preferred Reporting Items for Systematic Reviews and Meta‐Analyses Extension for Scoping Reviews) checklist was used to guide review methodology and reporting [26].

We operationally defined HCDs as any portable computer device that a person can hold in one hand and control using the other hand, including PDAs, smartphones, and tablet devices but excluding ubiquitous computing devices. Studies were screened against inclusion and exclusion criteria by one reviewer (DG). Ambiguous papers were subjected to full-text review. Two reviewers (DG and AH) independently performed full-text review of the screened papers, appraised the methodological quality of the included studies using Joanna Briggs Institute critical appraisal tools [27], and undertook data extraction. Disagreements about inclusion, quality appraisal, and data extraction decisions were resolved via consensus.

### Search Strategy

Keywords denoting HCDs varied among studies. Consequently, we used a strategy involving 3 successive literature searches (preliminary database search, broad search, and comprehensive search) to generate productive search terms and provide multiple patterns of literature coverage. All searches were limited to English language publications.

#### Literature Search 1—Preliminary Database Search

We undertook a preliminary scoping of literature published between 2001 and 2021 in CINAHL Complete, MEDLINE Complete, Embase, and Scopus. MEDLINE and CINAHL were searched using a combination of medical subject headings, CINAHL subject headings, and the following keywords: “Nursing Staff, Hospital”; “Acute Care Nurse Practitioner”; nurs*; “Decision Making, Computer Assisted”; “Decision Making, Clinical”; “Decision Making”; “Decision Making, Patient”; “Nursing Care Plans, Computeri?ed”; “clinical judgement”; “mobile application”; and “mhealth.” Embase and Scopus searched with simple keywords: “nurs*”; “decision*”; and “handheld computer*.” Search terms were initially derived from the systematic literature review by Mickan et al [9] and are reported in full in Multimedia Appendix 1 [6,28-54].

#### Literature Search 2—Broad Database Search

Results from the preliminary database search identified few studies on current generation HCDs such as smartphones, Android, iOS, and tablet devices. Therefore, a second search using a small number of broad search terms and limited to 2010 to 2021 was conducted to help identify additional keywords. We conducted this search in CINAHL Complete, MEDLINE Complete, Embase, and Google Scholar using the following keywords: “Decision Making, Computerised”; “nurs*”; “acute”; and “handheld” (Multimedia Appendix 1). It was noted that the reference lists of many relevant articles found in search 2 contained publications from the journal “CIN: Computers, Informatics, Nursing” that were not retrieved from the bibliographic databases. Consequently, we hand searched the articles published in this journal from 2010 to 2021.

#### Literature Search 3—Comprehensive Database Search

A final, comprehensive search was conducted in MEDLINE, Embase, and CINAHL (2010 to 2021). This search was conducted to determine whether a more structured and detailed search strategy derived from the results of literature searches 1 and 2 would identify a more comprehensive list of relevant studies. Details of the search algorithm is provided in Multimedia Appendix 1.

### Study Inclusion and Exclusion Criteria

Studies were included according to the following criteria: (1) primary research study; (2) study that explored the effect, acceptability, or usability of HCDs with respect to nurses’ clinical decision-making; (3) study conducted in an acute care setting; and (4) peer-reviewed journal publication. Conference proceedings and dissertations, studies that did not include nurses in the evaluation, studies not conducted at the point of patient care, and studies that explored the effect of HCD devices on nurse education or professional development were excluded from the review. As this was a scoping review, studies were not excluded on the basis of study design or methodological quality.

### Data Extraction and Data Analysis

Management of scoping review citations and study data was undertaken in the Covidence reference management platform [55]. To categorize studies according to their objectives, they were analyzed thematically. Two researchers (DG and AH) reviewed the selected papers and devised independent coding frames based on the emergent themes. The 2 researchers then discussed and refined the themes identified across the included studies until consensus on the final thematic structure was reached.

Study data were extracted and recorded on a spreadsheet, capturing study characteristics—design, methodology, sample size context, type of computer technology used, information delivery mode, decision support information provided—and study outcomes—outcome measures and findings.

## Results

### Number of Studies Identified by the Review

**Figure 1:**
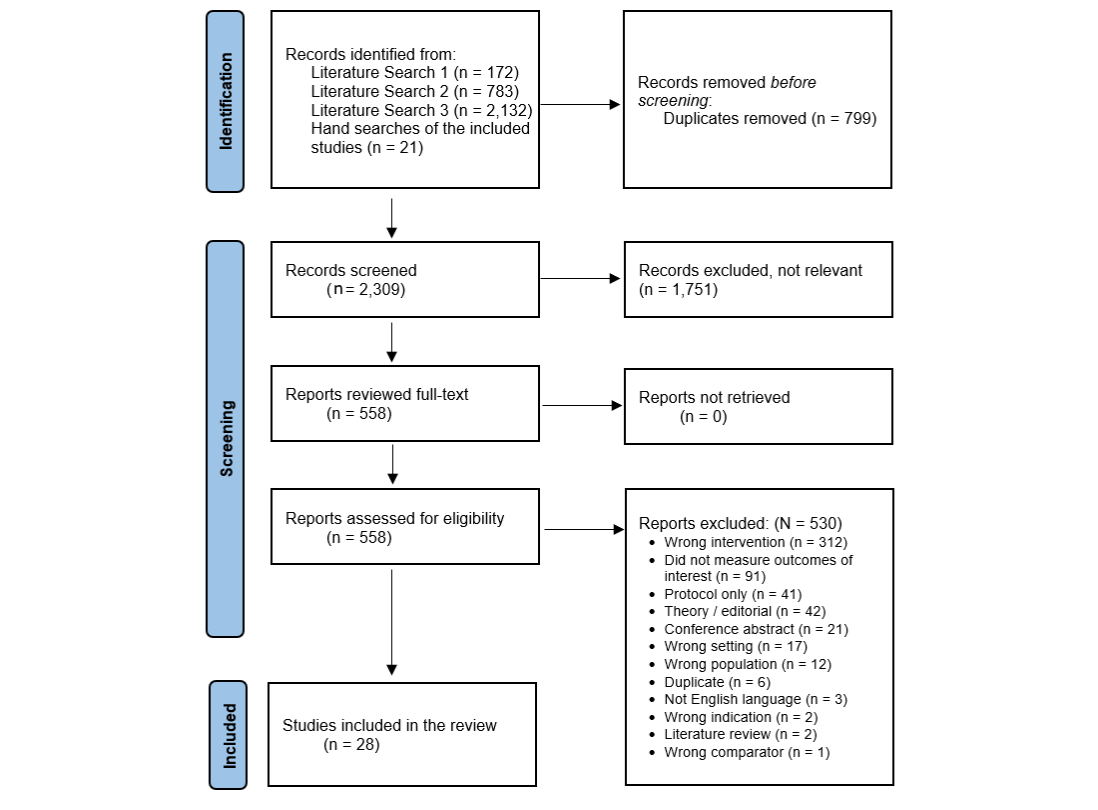
PRISMA (Preferred Reporting Items for Systematic Reviews and Meta-Analyses) flow diagram for literature searches 1-3.

A total of 3108 records were identified from the 3 literature searches and hand searches of reference lists. After removing duplicate records, 74.29% (2309/3108) of studies were screened based on title and abstract, and 24.17% (558/2309) underwent full-text review. A total of 98.79% (2281/2309) of studies failed to meet the inclusion criteria and were excluded from the review. Figure 1 reports the number of studies identified, screened, and included in this scoping review.

In total, 28 studies were included in the final analysis. These comprised randomized controlled trials (3/28, 11%) [28-30]; quasi-experimental studies (9/28, 32%) using nonequivalent [31], crossover [32,33] and before-after designs with [34,35] and without [36-39] controls; observational studies (10/28, 36%) involving prospective [40,41], retrospective [42] and cross-sectional [43-49] designs; qualitative descriptive studies (2/28, 7%) involving in-depth [50] and focus group [6] interviews; and studies of diagnostic accuracy (2/28, 7%) [51,52]. In addition, 7% (2/28) of mixed method studies were included in the review [53,54]. Owing to their prominent qualitative component, these studies were quality appraised using the Joanna Briggs Institute structured checklist for qualitative studies [27]. All judgments of the risk of study bias made using the Joanna Briggs Critical Appraisal Tools (2021) [27] are detailed in Multimedia Appendix 1.

Overall, 3 thematic areas of inquiry emerged from the included studies (Table 1): impact on clinical decision-making (12/28, 43%); enhancing the efficiency, safety, and quality of care (9/28, 32%); and handheld device usability, uptake, and acceptance (14/28, 50%). A total of 18% (5/28) of studies evaluated the use of older computerized decision support technology available on PDA devices [35,43,45,47,53]. Most (21/28, 75%) studies evaluated the use of digital applications delivered by modern smartphone or tablet devices, and 4% (1/28) evaluated the use of applications on both PDA and tablet devices [39]. The remaining (1/28, 4%) study evaluated the use of a mobile nursing information system that could be accessed at the bedside [54]. Overall, HCDs were described in the included studies using 24 unique terms covering 17 individual concepts (Table 2).

**Table 1:** Themes identified from the included studies.

Author, year	Impact on clinical decision-making				Enhancing the efficiency, safety, and quality of care				Handheld device usability, uptake, and acceptance				
	Identifies elements	Quantifies elements	Synthesizes elements	Identifies elements		Quantifies elements	Synthesizes elements	Identifies elements		Quantifies elements		Synthesizes elements	
Bakken et al [28], 2014	✓	✓											
Cato et al [41], 2014								✓					
Cleaver et al [31], 2021	✓												
Doran et al [39], 2010								—^a^		—		—	
Farrell [6], 2016				✓				✓					
Godwin et al [32], 2015	✓	✓											
Hsiao and Chen [43], 2012								—		—		—	
Johansson et al [34], 2012				✓				✓					
Johansson et al [44], 2014				—		—	—	—		—		—	
Kartika et al [52], 2021	✓	✓											
Kerns et al [42], 2021	✓	✓	✓										
Lin [45], 2014				✓									
McCulloh et al [46], 2018								✓		✓			
Momtahan et al [53], 2007								✓		✓		✓	
Moore and Jayewardene [48], 2014								—		—		—	
O’Donnell et al [51], 2019	✓	✓	✓					✓		✓		✓	
Reynolds et al [54], 2019				✓		✓		✓		✓			
Ricks et al [50], 2015				✓		✓							
Ruland [35], 2002	✓												
Sedgwick et al [37], 2017	✓												
Sedgwick et al [38], 2019	✓			✓									
Sefton et al [33], 2017	✓	✓	✓					✓		✓		✓	
Siebert et al [29], 2017				✓		✓							
Siebert et al [30], 2019				✓		✓							
Shen et al [47], 2018								—		—		—	
Singh et al [36], 2017	✓	✓	✓										
Spat et al [40], 2017	✓	✓											
Yuan et al [49], 2013								✓			✓		✓

^a^Specific handheld computer device intervention not included in research or indicated by authors.

**Table 2:** Descriptors for “handheld computer device” in the included studies (N=28).

Term			Frequency, n (%)		References
**PDA technology (n=6, 21%)**					
	PDA	3 (11)		Satterfield et al [10], Shen et al [47], and Momtahan et al [53]	
	Mobile nursing information system	2 (7)		Hsiao and Chen [43] and Lin [45]	
	Handheld technology	1 (4)		Ruland [35]	
**Smartphone or tablet technology (n=22, 79%)**					
	Mobile device application or mobile device app or mobile-based application	3 (11)		Siebert et al [29], Siebert et al [30], and Kartika et al [52]	
	Mobile computerized decision support system or mobile computing devices	2 (7)		Spat et al [40] and Ricks [50]	
	Mobile devices or advanced mobile devices	2 (7)		Johansson et al [34] and Johansson et al [44]	
	Mobile electronic clinical decision support or mobile device–based electronic decision support tool	2 (7)		McCulloh et al [46]	
	Mobile health decision support system	2 (7)		Bakken et al [28] and Cato et al [41]	
	Mobile technologies	2 (7)		Sedgwick et al [37] and Sedgwick et al [38]	
	Smartphone or iPhone	2 (7)		Farrell [6] and Moore and Jayewardene [48]	
	Tablet PC or Android tablet	2 (7)		Doran et al [39] and O’Donnell et al [51]	
	Clinical decision support system	1 (4)		Yuan et al [49]	
	Electronic physiological surveillance system	1 (4)		Sefton et al [33]	
	Smart device–based application	1 (4)		Godwin et al [32]	
	Tablet app	1 (4)		Cleaver et al [31]	
Health care–specific technology (handheld decision support device)			1 (4)		Reynolds et al [54]

### Impact of Handheld Device Use on Clinical Decision-making

The characteristics and outcomes of the 12 studies that investigated HCDs as clinical decision-making supports are summarized in Table 3. The studies in this group investigated the extent to which HCDs could improve the quality of clinical assessment and management decisions or processes (10/12, 83%) [28,31-33,35,36,40,42,51,52] and enhance nurses’ capacity for clinical decision-making (2/12, 17%) [37,38]. HCD interventions targeted processes of nurse clinical decision-making using a range of modalities, which varied according to the predominance of clinician versus algorithmic judgment. These were as follows: (1) clinical reference guides to support unstructured clinical judgments (2/12, 17%) [37,38]; (2) aide-mémoire to structure clinical judgment (3/12, 25%) [28,31,35]; and (3) fully computerized algorithmic assessments [32,52], drug dosing [40], and clinical pathways [33,36,42,51], with varying levels of clinician override (7/12, 58%). In total, 8% (1/12) of studies investigating the impact of HCD use on clinical decision-making used PDAs [35], whereas the remaining 92% (11/12) of studies used modern HCD technology. Studies within this theme were undertaken in emergency departments (4/12, 33%) [31,36,42,51], in various health and hospital settings (3/12, 25%) [28,37,38], in inpatient wards (3/12, 25%) [35,40,53], in an infectious pediatric ward (1/12, 8%) [52], and in the context of a laboratory-based simulation (1/12, 8%) [32].

Research investigating the impact of HCDs on the quality of clinical decisions or processes (10/28, 36%) found significantly greater rates of diagnosis when using an HCD system for the assessment and management of obesity, tobacco use, and depression [28]; improved prediction of serious illnesses [33] and clinical deterioration in hospitalized children using HCD-based assessment instruments [52]; more consistent nursing documentation after the implementation of an HCD support system for patients’ care plans and preferences [35]; lower odds of hospital admission and shorter length of hospital stay in pediatric patients and increased referrals to smoking cessation programs in child caregivers from computerized asthma management support [42]; faster calculation of body surface area and intravenous fluid replacement rates in patients with burns when using an automated tool [32]; and faster treatment decision-making [31] and increased patient confidence [36] via the use of tablet-based emergency department assessment applications. One study reported that an Android tablet tool was able to successfully identify patients who required an electrocardiogram within 10 minutes of presentation [51]. Finally, the use of computerized decision support for insulin dosing in type 2 diabetes yielded a high level of agreement with standard clinical assessments (97%) and was perceived to precipitate a reduction in treatment decision-making errors [40].

Research (2/28, 7%) examining the degree to which handheld technology may enhance nurses’ self-rated decision-making capacity reported no significant impact from HCDs. Specifically, Sedgwick et al [37] found that the use of a nursing smartphone app (“PEPID”) over the course of 1 month failed to significantly improve nurses’ ratings of self-efficacy and the ability of new graduate nurses to make clinical decisions in a rural hospital setting. A later publication by the same authors [38] reported that the intervention was not associated with nurses’ perceptions of improvements in their clinical decision-making processes. However, notably both studies had small sample sizes (n=25 and n=20), which limited their statistical power.

**Table 3:** Characteristics of the included studies that explored clinical decision-making.

Reference	Theme (subtheme)	Study setting	Type of intervention	Outcome measure	Study type	Participant, n	Results
Bakken et al [28], 2014	Quality of clinical decision-making (*assessment or care decisions*)	Various health settings	Decision support (aide-mémoire for structured clinical judgment): handheld decision support tool for the assessment and management of obesity, tobacco use, and depression using screening prompts, standardized screens, selection of patient goals, clinical practice guidelines, and recording treatment plans	Number of encounters with a clinical practice guideline–related diagnosis Number of care plan items in encounters with a clinical practice guideline–related diagnosis.	Randomized controlled trial	363 registered nurses undergoing nurse practitioner education	Significant effect of the intervention on diagnostic rates
Cato et al [41], 2014	Usability, uptake, and acceptance (*predictors of HCD^a^ use*)	Acute and ambulatory care settings in the New York City metropolitan area	Decision support (aide-mémoire to initiate screening and select treatment): tobacco cessation screening and treatment prompt housed on mobile device or devices	Number of encounters resulting in nurse screening for tobacco use, provision of smoking cessation advice, and patient referrals for smoking cessation treatments	Observational study of the intervention arm of a randomized controlled trial	14,115 patient encounters involving 185 registered nurses	Screening was more likely in patient encounters involving women (OR^b^ 1.14, 95% CI 1.03-1.25) or African American patients (OR 1.18, 95% CI 1.01-1.38) Screening was higher in patients cared for by specialty nurses (OR 4.43, 95% CI 3.20-6.13) or in sites where the predominant payer was Medicare, Medicaid, or SCHIP^c^ (OR 1.88, 95% CI 1.57-2.24). In these sites, nurses were more likely to provide tobacco cessation teaching and counseling (OR 1.74, 95% CI 1.03-2.94) and less likely to provide treatment referrals for tobacco cessation (OR 0.439, 95% CI 0.252-0.764). Patient encounters by nurses in FNP^d^ (OR 0.381, 95% CI 0.209-0.693) or PNP^e^ (OR 0.314, 95% CI 0.109-0.906) specialties were less likely to provide treatment referrals
Cleaver et al [31], 2021	Quality of clinical decision-making *(assessment or care decisions)*	2 metropolitan hospital EDs^f^, London, United Kingdom	Decision support (aide-mémoire for structured clinical judgment): tablet-based decision support app to assist ED nurses to select investigations and treatments at initial patient assessment	Speed and accuracy of clinical decisions (including patient acuity score rating) compared with control. Nurse assessment and subsequent expert panel evaluation.	Retrieval and analysis of the stored device data on the type and time of requests made by nurses compared with control nurse decisions and independent postevent review by expert panel	Number of nurse participants was not specified; 529 patient assessments performed via app	Demonstrated time improvements in the identification and actioning of appropriate patient investigations, treatments, and procedures Need to improve some user design features identified
Doran et al [39], 2010	Usability, uptake, and acceptance (*patterns of use*)	29 acute, long-term, home care, and correctional organizations, Ontario, Canada	No specific intervention—survey of staff perceptions of technology use: mobile devices, including PDAs and tablet computers	Perceived impact of the mobile technologies on the barriers to research use, quality of care, and job satisfaction	Prestudy and poststudy questionnaires	488 frontline nurses	Over 44.5% of the nurses used mobile device at least once every few days
Farrell [6], 2016	Enhancing efficiency, safety, and quality of care (*clinical or interdisciplinary communication*) Usability, uptake, acceptance (*assess usability or identify heuristics or human factors or ergonomic considerations*)	Acute gynecological ward, Melbourne, Australia	Electronic clinical reference guide: iPhone with clinical resource and medication information apps—use by nurses in the acute care setting	To explore nurses’ perspectives on iPhone use within an acute care unit	Not available	20 registered nurses	iPhones were accessible and portable and enhanced workplace communication Negative findings: small screen size inhibited use, especially for patient teaching, and device use was perceived to be unprofessional in direct patient care setting
Godwin et al [32], 2015	Quality of clinical decision-making (*assessment or care decisions*)	Laboratory study	Computerized measurement tool: a software app for Apple devices that facilitates the calculation of total BSA^g^ of patients with burns. App also includes a fluid replacement formula ready reckoner and serial wound photography platform.	Accuracy of app versus traditional “longhand” calculation tools	Repeat measure observation, with 1 week washout between method testing by participants	11 health clinicians, including ED nurses	App allowed faster calculation of BSA and fluid requirements and wound type evaluation compared with traditional methods, with no loss of accuracy
Hsiao and Chen [43], 2012	Usability, uptake, and acceptance (*quality of data entered or retrieved*)	Regional Hospital, Taiwan	No specific intervention—survey of staff perceptions of technology use: “m-NIS” available on PDA, notebook, or “panel” computer	Factors affecting fit between mobile nursing system and nursing tasks, task-technology fit, and nursing performance	Prestudy and poststudy questionnaires	310 clinical nurses recruited, with 210 questionnaires returned	Positive effect on information acquisition, integration, and interpretation in nursing
Johansson et al [34], 2012	Enhancing the efficiency, safety, and quality of care (*impact on activity flow and perceived safety*) Usability, uptake, and acceptance (*quality of data entered or retrieved*)	Orthopedic ward, palliative care unit, and rural district hospital in Norway	Electronic clinical reference guide: use of mobile phones in clinical nursing practice for 15 weeks	To explore the mobile device’s usefulness in terms of information retrieval, ability to save time, patient safety, quality of care, and work confidence	Descriptive prestudy and poststudy written surveys	Registered nurses (n=14) and nursing students (n=7)	Mobile device perceived as useful and time saving. It also contributed to improved patient safety and quality of care by improving access to information
Johansson et al [44], 2014	Enhancing the efficiency, safety, and quality of care (*impact on activity flow*) Usability, uptake, acceptance (*quality of data entered or retrieved*)	Multiple health care agencies, Sweden	No specific intervention—survey of staff perceptions of technology use: the use of mobile devices	Views regarding the use of advanced mobile devices in nursing practice	Cross-sectional survey	62 graduate nurses working in acute care settings (*of a larger sample of 107 nurses*)	Participants regarded an advanced mobile device to be useful for accessing resources, making notes, planning their work, and saving time
Kartika et al [52], 2021	Quality of clinical decision-making *(assessment or care decisions)*	Infectious pediatric ward of a major referral hospital, Indonesia	Computerized risk assessment tool: mobile computing application to assess the risk of clinical deterioration, mPEWS^h^-InPro	AUC^i^ or ROC^j^ Sensitivity and specificity of cut points	Test of diagnostic accuracy	108 pediatric patients	Analyses indicated that the mPEWS-InPro had a strong predictive ability: AUC=0.942 (95% CI 0.865-1.000; *P*=.001) Using a cut point of 4, the mPEWS-InPro had a sensitivity of 92.3% and a specificity of 80%
Kerns et al [42], 2021	Quality of clinical decision-making (*assessment or care decisions*)	Emergency and inpatient departments in 75 freestanding Children’s or community Hospitals in the United States	Decision support (algorithmic clinical pathways): mobile “mECDS tool” that provided evidence-based clinical support for the management of pediatric asthma	Determine the impact of the tool on pediatric asthma care quality	Observational study (digital review of screen use by practitioners)	Tool used on 286 devices and 355 times for 4.191 digital events (approximately 50:50 access events in ED versus inpatient settings)	Significantly reduced odds of hospital admission through the use of the mECDS^k^ tool Higher rates of referrals to smoking cessation programs in caregivers through the use of the tool Shortened hospital length of stay
Lin [45], 2014	Enhancing the efficiency, safety, and quality of care (*clinical or interdisciplinary communication*)	Major regional medical center, Taiwan	Electronic clinical reference guides: a mobile nursing “Cart,” PDA, and tablet device providing access to an m-NIS^l^ program (details of this program were not provided)	Factors affecting the fit between nursing tasks and mobile nursing information systems and nurse performance from the perspective of task-technology fit	Postimplementation questionnaire	219 surveys returned	m-NIS improved message exchange between health care professionals and communication with patients, increased the efficiency of patient care duties, improved the quality of care, increased the professional image of nursing, and im- proved the overall performance in nursing practices
McCulloh et al [46], 2018	Usability, uptake, and acceptance *(patterns of use)*	Inpatient pediatric settings, United States	Decision support (*algorithm for structured clinical judgment*): smartphone-based evidence-based “PaedsGuide” electronic decision support tool	Tool development, distribution, and use patterns	Descriptive analysis (*data analytics and web-based user feedback survey*)	3805 multidisciplinary health care practitioner users (*number of nurses was not specified*)	Of the total user screen time, 61% was spent viewing clinical practice benchmarks, including hospital admission appropriateness, length of hospitalization, and diagnostic testing recommendations Positive feedback on tool’s usability
Momtahan et al [53], 2007	Usability, uptake, and acceptance (*assess usability or identify heuristics or human factors or ergonomic considerations*)	Canadian acute heart center	Decision support (algorithmic clinical pathways): PDA cardiac patient symptom decision support tool	Viability and value of the digital handheld decision support tool compared with standard paper-based survey approach (*retrospective cardiologist opinion on nurse evaluation*)	“Cognitive work analysis” and semistructured interviews following 3-month trial	9 cardiac nurse coordinators	Data collection was more complete and clearer with PDA assessment Nurses found the PDA tool more helpful than the paper-based tool Cardiologists concurred with nurse assessment outcomes in 97% of cases
Moore and Jayewardene [48], 2014	Usability, uptake, and acceptance (*patterns of use*)	161 acute NHS^m^ trusts	No specific intervention—survey of staff perceptions of technology use	Questionnaire measuring the patterns of app use, factors affecting app use, and perceived effects on patient care	Cross-sectional survey	82 nurses and 334 doctors	Participant responses indicated a large number of users of textbooks, formularies, clinical decision tools, and calculators.
O’Donnell et al [51], 2019	Quality of clinical decision-making (*assessment or care decisions*) Usability, uptake, and acceptance (*assess usability or identify heuristics or human factors or ergonomic considerations*)	Hospital emergency department, Dublin, Ireland	Decision support (algorithmic clinical pathways): Android tablet tool (AcSAP^n^) determining the probability of patients with suspected coronary syndrome, prompting ECG^o^ performance on patients within 10 minutes	Efficacy of the app in identifying patients requiring an ECG Time until performance of ECG	Patient history audit of time of presentation, triage action, and first ECG and diagnosis and postuse questionnaire on app usability	AcSAP app was activated 379 times by triage nurses (exact number of nurses unstated). 18 triage nurses returned the postuse questionnaire	App successfully identified patients who required an ECG within 10 minutes of presentation App assessed as easy to use by the participants
Reynolds et al [54], 2019	Enhancing the efficiency, safety, and quality of care *(perceived safety)* Usability, uptake, and acceptance *(assess usability or identify heuristics or human factors or ergonomic considerations)*	Neonatal and pediatric intensive care units across 2 hospitals in California, United States	Medication dosing support: nurse use of stand-alone customized handheld drug and IV^p^ infusion calculation aid	User acceptance and effect of the device	Mixed methods: ethnographic observation, prestudy and poststudy interviews, and surveys	64 nurses	Device was perceived to be worthwhile; risk perceptions and device usability limited device use No significant difference in cognitive load or administration errors
Ricks et al [50], 2015	Enhancing the efficiency, safety, and quality of care *(nurses’ perceptions of care quality)*	Public hospital in Port Elizabeth, South Africa	Electronic clinical reference guide and medical calculator: nurses’ use of a smart phone device at the point of care to access electronic resources, namely a disease directory, drug list treatment guidelines, and a medical calculator	To explore the experiences of registered nurses in using the device	Qualitative descriptive study	A total of 50 nurses; purposive sampling of 10 nurses for in-depth interview	The device improved computer literacy, was useful for patient and in-service education, improved the accuracy of diagnosis, increased practice delivery and, improved the quality of care
Ruland [35], 2002	Quality of clinical decision-making *(assessment or care decisions)*	Acute medical care unit in Oslo, Norway	Decision support (aide-mémoire for structured clinical judgment): “Palm-pilot” handheld computerized support system (“CHOICE”) that assists nurses to determine patient preferences to incorporate into the care plan	Effects of the system on nurses’ care priorities and preferences and patient satisfaction	3 group sequential survey design	28 nurses	Use of the system resulted in improved consistency between patients’ and nurses’ care preferences
Sedgwick et al [37], 2017	Quality of clinical decision-making *(capacity for clinical decision-making)*	Rural hospital, Lethbridge, Canada	Electronic clinical reference guide: Tepid^q^ app (containing multiple nurse resources) on personal mobile device	Impact of mobile technologies on graduate nurses’ perceived decision-making abilities and self-efficacy	Quasi-experimental pretest and posttest design	A total of 25 graduate student nurses (on clinical placement) were recruited and 12 completed the full questionnaire	Use of app did not enhance self-perceived efficacy or decision-making ability
Sedgwick et al [38], 2019	Quality of clinical decision-making *(capacity for clinical decision-making)* Enhancing the efficiency, safety, and quality of care *(impact on activity flow)*	Rural hospital, Lethbridge, Canada	Electronic clinical reference guide: personal smartphone app “PEPID professional Nursing Suite App” (providing access to multiple clinical nursing resources)	Effect on nurses’ walking distance and clinical decision-making ability	Prestudy and poststudy surveys	20 clinical nurses	No significant reduction in nurses’ clinical walking distance No self-perceived effect on nurses’ decision-making ability Increased confidence in using app over time
Sefton et al [33], 2017	Quality of clinical decision-making *(assessment or care decisions)* Usability, uptake, and acceptance *(quality of data entered or retrieved)*	Pediatric hospital, United Kingdom	Computerized measurement tool with pathway decision support: handheld digital “Paediatric Warning System” tool to identify the development of serious illnesses (iPod Touch 4th generation [Apple Inc])	Accuracy of vital sign readings and time taken to document compared with paper-based method	Prospective mixed methods	A total of 23 RNs^q^, student nurses, health service attendants, and medical students	Improved documentation speed, accuracy, and clarity with the use of the digital device
Shen et al [47], 2018	Usability, uptake acceptance *(quality of data entered or retrieved)*	Various (nonspecified) clinical departments of a major tertiary hospital in Beijing, China	No specific intervention—survey of staff perceptions of technology use: PDA providing access to mobile nursing information system	Clinical nurses’ satisfaction with the use of PDA	Cross-sectional descriptive survey	383 nurses	Nurses were more satisfied with the delivery of medical orders and documentation facility of the device Utility was dependent on the stability of network, and higher satisfaction positively correlated with nurse education level
Siebert et al [29], 2017	Enhancing the efficiency, safety, and quality of care *(impact on activity flow)*	Pediatric emergency department, Switzerland	Medication dosing support: tablet-based app to support decision-making for the continuous infusion of medications	Drug preparation time, time of drug delivery, and number of medication errors	Randomized controlled crossover trial	20 nurses	Intervention significantly reduced drug preparation time, time to drug delivery, medication errors.
Siebert et al [30], 2019	Enhancing the efficiency, safety, and quality of care *(impact on activity flow)*	3 regional pediatric emergency departments in Switzerland	Medication dosing support: tablet-based app to support decision-making for continuous infusion of medications	Drug preparation time, time to drug delivery, number of medication errors	Randomized controlled crossover trial	128 nurses	Intervention significantly reduced drug preparation time, time to drug delivery, and medication errors
Singh et al [36], 2017	Quality of clinical decision-making *(assessment or care decisions)*	Emergency department, Connecticut, United States	Decision support (algorithmic clinical pathways): use of a bedside tablet computer app to assess patients and guide decisions on the performance of a CT^r^ scan in patients with concussion	Effects of the tool on patient experience, clinician experience, health care use, and patient safety	Pilot study with prestudy and poststudy surveys of patient and clinician experiences	A total of 2 advanced practice nurses, 16 physicians, 11 physician assistants (41 patients enrolled)	The trust of the patients in their physicians increased when they were satisfied with the use of the tool and the clarity of information it provided; most clinicians perceived the app to be helpful for patients and to be usable No clinically important brain injury was missed through the use of the device
Spat et al [40], 2017	Quality of clinical decision-making *(assessment or care decisions)*	General hospital ward, Graz, Austria	Medication dosing support: customized Samsung Galaxy tablet (Samsung Group) computer designed to assist nurses and medical officers in determining the appropriate insulin dose for patients with type 2 diabetes	Safety, efficacy, and user acceptance of device or system	Feasibility study using field notes on use and prestudy and poststudy written questionnaires	At time point one, 14 nurses and 12 physicians had participated; at time point 2, 12 nurses and 3 physicians had participated and at time point 3, 12 nurses and 6 physicians had participated	High use of device, high confidence in the use of tool over time, and high level of device decisions agreement by health care providers (97%) Perceptions that treatment errors reduced through the use of the device
Yuan et al [49], 2013	Usability, uptake, and acceptance *(assess usability or identify heuristics or human factors or ergonomic considerations)*	Hospital setting, Texas, United States	Decision support (algorithmic clinical pathways): bedside clinical decision support system housed on tablet devices	Number of heuristic violations Number of successful case simulations Duration of the simulated task NASA^s^ Task Load Index	Heuristic evaluation	A panel of evaluators comprising 3 licensed vocational nurses and 7 registered nurses	Simulation sessions resulted in the following: 83 heuristic violations 100% of successful completions (n=30 sessions) An average of 111 (SD 30) seconds to complete the simulated task NASA Task Load Index results indicated low cognitive and physical burden

^a^HCD: handheld computer device.

^b^OR: odds ratio.

^c^SCHIP: State Children’s Health Insurance Program.

^d^FNP: family nurse practitioner.

^e^PNP: pediatric nurse practitioner.

^f^ED: emergency department.

^g^BSA: body surface area.

^h^mPEWS: Modified Pediatric Early Warning System.

^i^AUC: area under the receiver operating characteristic curve.

^j^ROC: receiver operating characteristic.

^k^mECDS: mobile electronic clinical decision support tool.

^l^m-NIS: mobile nursing information system.

^m^NHS: National Health Service.

^n^AcSAP: acute coronary syndrome application.

^o^ECG: electrocardiogram.

^p^IV: intravenous.

^q^RN: registered nurse.

^r^CT: computed tomography.

^s^NASA: National Aeronautics and Space Administration.

### Enhancing the Efficiency, Safety, and Quality of Care

The characteristics and outcomes of the 32% (9/28) of studies that investigated the effect of HCD decision support systems on the efficiency, quality and safety of care delivery are summarized in Table 3. Studies that addressed this theme examined the impact of HCDs on the flow of nursing activities (5/9, 56%) [29,30,34,38,44], team communication (2/9, 22%) [6,45] and care safety (2/9, 22%) [34,54] and explored nurses’ perceptions of the quality of their HCD-facilitated care in the clinical space (1/9, 11%) [50]. The research targeted two decision-making modalities: (1) unstructured clinical judgment via the use of electronic clinical reference guides with (1/9, 11%) [50] or without (4/9, 44%) [6,34,38,45] medical calculator support and (2) fully computerized algorithmic judgments for drug dosing (3/9, 33%) [29,30,54]. The authors of the remaining study (1/9, 11%) did not specify the type of mobile computing apps accessed by nurses [44]. Apart from studies involving both PDA and tablet technology [45] and one involving a stand-alone handheld drug and intravenous infusion calculator [54], this research investigated modern HCD technology. A range of acute care contexts was represented, including rural hospitals (2/9, 22%) [34,38]; pediatric emergency departments (2/9, 22%) [29,30]; and medical (1/9, 11%) [45], gynecological (1/9, 11%) [6], orthopedic (1/9, 11%) [34], palliative care (1/9, 11%) [34], and neonatal and pediatric intensive care (1/9, 11%) [54] units. Moreover, cross-sectional studies were conducted across multiple clinical environments in acute care [44,50].

Most (4/5, 80%) published research that examined the flow of nursing activities reported a positive association between the use of HCDs in the clinical setting and nursing efficiency. Research that found a positive clinical impact of HCDs in this respect comprised 50% (2/4) of studies of an HCD-based medical dosing support system, which resulted in significantly reduced drug preparation time, time to drug delivery, and medication errors compared with usual care [29,30] and 50% (2/4) of studies investigating the use of electronic clinical reference guides, which found that HCDs were associated with self-reported time saving [34,44]. The remaining (1/5, 20%) study [38] reported that the implementation of a nursing smartphone app did not significantly modify nurses’ work efficiency, as measured by the distance walked during each shift.

The literature suggested that HCD interventions enhanced team communication and were safe, but the number of relevant studies was less. Authors reporting on postimplementation focus groups [6] and questionnaire [45] findings reported that HCD-based electronic clinical reference guides improved nurse communication, and these interventions were also associated with nurse reports of increased quality and patient safety in acute care delivery [34]. A further study, which evaluated the safety of computerized medication dose calculations, reported that despite concerns regarding the perceptions of risk with respect to the HCD device, there were no significant differences in the rate of medication administration errors when using the intervention, relative to usual care [54].

The final study within the theme of efficiency, safety, and quality of care explored perceptions of HCD-facilitated care quality in 10 nurses [50]. Interviewees believed that the use of smartphone devices at the point of care with access to a disease directory, pharmacological treatment guidelines, and a medical calculator improved their diagnostic accuracy and quality of patient care. Smartphones were also said to improve nurses’ computer literacy skills and have utility in facilitating the delivery of patient and in-service education.

### Handheld Device Usability, Uptake, and Acceptance

Studies that evaluated the usability, uptake, and acceptance of HCDs among clinical end users are detailed in Table 3 (14/28, 50%). These studies sought to evaluate the quality of data entry into, or retrieval from, HCD platforms (5/14, 36%) [33,34,43,44,47]; assess usability with respect to heuristics, human factors, or ergonomics (5/14, 36%) [6,49,51,53,54]; describe patterns of HCD use in clinical staff (3/14, 21%) [39,46,48]; and identify predictors of the use of HCD interventions (1/14, 7%) [41]. Research within this theme investigated the usability, uptake, or acceptance of the following: (1) electronic clinical reference guides [6,34] or prompts [41] to supplement unstructured clinical judgments (3/14, 21%); (2) decision support algorithms to facilitate structured clinical judgment (1/14, 7%) [46]; and (3) algorithmic clinical pathways [33,49,51,53] and drug dosing [54] (5/14, 36%). The remaining 36% (5/14) of studies on this theme explored the use of mobile phone [39,44,48] and PDA [39,43,47] technology involving no prespecified HCD intervention.

Studies that explored HCD usability, uptake, and acceptance varied by technology platform. Except for 21% (3/14) of PDA studies [43,47,53] and 7% (1/14) of studies of a stand-alone handheld drug and intravenous infusion calculator, research on this theme examined modern smartphone and tablet technologies (10/14, 71%). Usability, uptake, and acceptance studies were undertaken in acute hospital settings [39,41,44,47-49], including an emergency department [51], a gynecological ward [6], a heart center [53], a regional hospital [43], orthopedic and palliative care units [34], and pediatric inpatient settings [33,46,54]. Studies by Cato et al [41] and Doran et al [39] also included patients from long-term, home care, and correctional organizations and ambulatory settings, respectively. Analyses in these studies were not stratified according to acute care status.

The reviewed findings consistently suggested that HCD devices may facilitate improved processes for clinical data entry and retrieval at the point of care (5/14, 36%). For example, recording children’s physiological data was found to be faster and more accurate when using a handheld device compared with traditional written medical records [33], and a further study revealed that nursing staff perceived the use of smartphone technology to improve their ability to access information [34,44], record notes, and plan care [44]. Finally, nurses perceived PDAs to assist the retrieval, integration, and interpretation of clinical data [43]. However, Shen et al [47] found that nurses’ perceptions of utility varied according to the stability of the wireless network and the level of their education. Specifically, the authors reported that nurses with more education and years of clinical experience were more satisfied with using the device.

Studies of the usability of HCD interventions with respect to heuristics, human factors, or ergonomics (5/14, 36%) reported varied findings. Cognitive work analysis interviews undertaken with cardiac nurse coordinators (9/14, 64%) suggested that the PDA-based decision support aids were easier to use and yielded clearer and more consistent data collection by nursing staff than their paper-based equivalents [53]. In another study, nurses described an Android-based app to aid decisions in suspected coronary syndrome as easy to use [51]. Despite these positive findings, other research highlighted barriers to HCD usability precipitated by device and design issues. These included the limitations of small size of the device screen and the perception that patients may view smartphone use in the clinical setting as unprofessional [6] and performance- and interface-related concerns that affected both time efficiency and nurses’ willingness to adopt the technology [54]. Studies reported that nurse who operated HCDs had low cognitive and physical burden [49] and no significant difference in cognitive load or administration errors in HCDs versus usual care [54].

Descriptive research on nurses’ patterns of HCD use (3/14, 21%) reported on the frequency of self-initiated access of mobile phones and PDAs in the clinical space [39,48] and identified the elements accessed in a pediatric electronic decision support tool [46]. Although the data suggested that it was common for nurses to use mobile devices and PDAs for clinical purposes [39,48], a study of the predictors of HCD use suggested that the adoption of HCDs may vary according to a combination of nurse, patient, and hospital characteristics [41]. The authors found that patient tobacco cessation screening was significantly more likely when nurses were advanced practice nurses; when the patients were women or African American; and when the predominant payer was Medicare, Medicaid, or the State Children’s Health Insurance Program.

## Discussion

### Principal Findings

The social pervasiveness of HCD technology, coupled with its low cost, provides nurses with access to a set of tools capable of optimizing patient care at the bedside. Despite the slow initial adoption of this technology [9], this systematic scoping review of the literature found that some studies of this technology have been conducted in this emerging area (n=28 studies). Positive impacts of HCD adoption were reported within the literature with a high degree of consistency. However, the low-to-moderate level of evidence characterized by observational and quasi-experimental designs and the dearth of studies that investigate the degree to which HCD implementation may disrupt the existing workflows limit the strength of conclusions drawn about the overall clinical impact of HCDs at the point of care.

This review identified that the literature investigating nurses’ use of HCDs at the point of care has targeted a variety of decision-making modalities ranging from the use of static guides supporting unstructured clinical judgments to fully algorithmic decisions based upon the computation of patient characteristics. Presently, the prevailing models of the psychology of decision-making identify 2 qualitatively distinct types of mental processing for decision-making: type 1, “autonomous processing,” reflecting automatic, rapid, intuitive, or associative judgments; and type 2, “effortful processing,” reflecting conscious or slow processing (ie, logical or hypothetical thinking) [56,57]. Structured clinical judgment and fully computerized judgment reduced or removed clinician input and, thus, the potential influence of cognitive biases typically associated with type 1 thinking [58]. Notably, a very large volume of empirical research has identified the superiority of algorithmic versus human judgment [13-15]. Consequently, it seems likely that these mechanisms at least partially account for the finding that the HCD interventions that formally structured or directed clinical judgment typically reported more positive outcomes than usual care.

Although the current trend of implementing HCD support for more structured nursing applications may better leverage the available mobile computing capabilities, many nursing tasks require rapid clinical judgments based on clinical experience [59]. Clinical reasoning is considered by professional nursing organizations to be fundamental to the very role of the nurse [60-62]. However, at present, research offers little to support the effectiveness of, or strategies for, HCD supports for nurses’ routine workflows. Aside from studies that duplicated the existing paper-based clinical information into an electronic format or allowed nurses to use HCDs to access the electronic resources they wanted, there has been a lack of research into how HCDs could be used to support nurses’ routine work outside the narrowly defined, technical nursing tasks investigated. Although the findings did generally indicate improvement within these discrete areas, the assortment and availability of HCD applications appears to be rather piecemeal such that the literature offers little guidance regarding the generalizability of these findings to other settings, total integration of individual applications within hospital information systems, or greater integration of HCD technology into nurses’ workflow. Thus, at present, the empirical literature does not provide clarity on the worth and utility of HCD technology in nursing work, and its transformative potential remains unclear.

This review found that the most frequently undertaken domain of study on the bedside use of HCDs concerned device usability, uptake, and acceptance. However, these issues have not been explored in depth, or study outcomes were specific to individual interventions with unclear generalizability to external health settings. There was a high degree of reliance upon subjective outcomes such as staff self-reports, which may result in biased outcomes because of perceived pressures to respond positively. Although user uptake was a key component of several individual intervention studies, to date, the published research has not identified principle-based barriers and facilitators capable of guiding future HCD interventions. Furthermore, a serious gap in the existing research was the absence of detailed investigation into the degree to which HCD implementation may disrupt the existing workflows. Additional work in this area is critical to developing a more holistic understanding of the clinical value of HCD interventions to nursing care delivery in the acute health care setting.

Finally, this review identified significant heterogeneity in the descriptors used within the published literature to denote HCDs. Individual descriptors (n=24) could be subsumed under 3 discrete labels with respect to the type and degree of technological development (“PDA technology,” “Smartphone/tablet technology,” and “Health care–specific technology”). Despite this, there was limited uniformity between the descriptors *within* these overarching labels, demonstrating a lack of standardized terminology. To assist future discovery and categorization of studies in this field, efforts to standardize the language may prove fruitful. Initially, we recommend that future researchers include clear terms such as “smartphone” and “tablet” that readily communicate the technology platform being used.

### Recommendations for Future Research

The finding that the level of evidence within the body of empirical literature was insufficient to support meta-analyses indicates the critical need for additional research to investigate the impact of HCDs on clinical nursing care in the acute practice setting. Notably, the preponderance of small-scale studies using observational designs highlighted the need for large, well-designed experimental trials using randomization or cluster randomization, where possible. Furthermore, as much of the extant literature has evaluated the impact of HCDs in pooled samples of multidisciplinary health care cohorts, there is a need to measure nurse-specific outcomes via nursing-specific studies or multidisciplinary studies using stratified analyses. Finally, as the use of HCDs in nursing practice implicitly lends itself to data capture via large-scale digital connectivity, future investigations in this field should attempt to leverage the potential of “big data” involving sizable data sets from multiple users across multiple domains [63]. Despite the conceptual and technical challenge presented, there should also be an exploration of the degree to which applications using Bayesian or machine learning techniques could support nurses’ clinical judgments.

Many of the retrieved studies measured the nursing and patient impacts of digital tools focused on multidisciplinary health assessment, diagnosis, and treatment modalities. However, this highlighted gaps in HCD-–based applications designed to support decision-making for other nurse-sensitive outcomes, including the assessment of clinical deterioration, patient comfort, functional status, and predischarge self-efficacy. Moreover, given the dynamic, treatment-based focus of care delivery in the acute health care environment, there is also a need to develop digital tools that support decisions for nursing care organization, including patient care prioritization, workflow, and safety. Other aspects of clinical handheld device use also need further exploration, including the potential benefits to patient care quality and safety resulting from productivity gains and the point-of-care use of specific computerized resources. Research should also systematically test the utility of various handheld programs’ user interface and interactivity designs to ensure that they assist rather than impede the flow of care delivery. Research should be undertaken to guide the future development of context-specific clinical HCD applications to improve the utility, safety, and value of such assistive devices in terms of the particular requirements and demands of acute nursing care delivery.

### Limitations

This scoping review had several limitations. First, as the primary focus was on published peer-reviewed literature, the gray literature was not comprehensively searched, and this may be an area of inquiry for future research. This would assist in determining the degree of positive publication bias present in the peer-reviewed literature. Second, this review was limited to studies undertaken in acute settings. Future research should investigate the degree to which impacts measured in nonacute settings may be generalizable and applicable across a range of health care settings. Third, this review was undertaken in the context of research for a minor thesis, without the resource to translate and include non-English publications.

### Conclusions

This paper has described the complexities involved in conducting a systematic scoping review and the dearth of quality research on the use of HCDs to support acute clinical nursing practice, highlighting the need for more targeted and rigorous research on this phenomenon. It is suggested that future research adopts a recognized nurse- and patient-sensitive outcome framework and focuses explicitly on the integration of mobile computing technology into the existing workflows and investigation of the impact of HCDs on patient care outcomes.

## Appendix

Multimedia Appendix 1 Summary data tables and annotated bibliography.

## Abbreviations

HCD: handheld computer device
PRISMA-ScR: Preferred Reporting Items for Systematic Reviews and Meta‐Analyses Extension for Scoping Reviews
