# Supplementary material for: Handheld Computer Devices to Support Clinical Decision-making in Acute Nursing Practice: Systematic Scoping Review
Source: J Med Internet Res. 2023 Feb 13;25:e39987. doi: 10.2196/39987 (PMC9972202; doi:10.2196/39987)
Supplement: Multimedia Appendix 1 [file jmir_v25i1e39987_app1.docx]

Online Supplemental Material

Online Supplement A – Literature Search 1 algorithm

| **CINAHL & MEDLINE (EBSCO) Search Strategy (Search conducted on September 19^th^, 2019)** |
| --- |
| 1. MH “Computers, Hand-Held” 2. TI “handheld Computer*” OR “AB “Handheld computer*” 3. TI “handheld device*” OR AB “handheld device*” 4. TI “handheld-device*” OR AB “handheld-device*” 5. TI iphone* OR AB iphone* 6. TI i-phone* OR AB i-phone* 7. TI smartphone* OR AB smartphone* 8. TI smart-phone* OR AB smart-phone* 9. TI “cellular phone*” OR AB “cellular phone*” 10. TI ipad* OR AB ipad* 11. TI i-pad* OR AB i-pad* 12. TI “tablet device*” OR AB “tablet device*” 13. TI “tablet computer*” OR AB “tablet computer* 14. TI “tablet PC*” OR AB “tablet PC*” 15. TI “tablet technolog*” OR AB “tablet technolog*” 16. TI mhealth OR AB mhealth 17. TI m-health OR AB m-health 18. TI “mobile health” OR AB “mobile health” 19. TI PDA* OR AB PDA* 20. TI “Personal digital assistant*” OR AB “Personal digital assistant*” 21. TI “touch screen device*” OR AB “touch screen device*” 22. TI “mobile W3 app*” OR AB “mobile W3 app*” 23. TI “Mobile W3 application*” OR AB “mobile W3 application*” 24. TI palmtop* OR AB palmtop* 25. S1 OR S2 OR S3 OR S4 OR S5 OR S6 OR S7 OR S8 OR S9 OR S10 OR S11 OR S12 OR S13 OR S14 OR S15 OR S16 OR S17 OR S18 OR S19 OR S20 OR S21 OR S22 OR S23 OR S24 26. MH “Acute Care Nurse Practitioner*” 27. MH “Nursing Staff, Hospital” 28. TI “Advanced Practice Nurse*” OR AB “Advanced Practice Nurse*” OR TI nurs* OR AB nurs* 29. S26 OR S27 OR S28 30. MH “Decision Making, Computer Assisted” 31. MH “Decision Making, Clinical” 32. MH “Decision Making” 33. MH “Decision Making, Patient” 34. TI ‘Clinical Judgement” OR AB “Clinical Judgement” 35. MH “Nursing Care Plans, Computeri?ed” 36. MH “Nursing Care Plans” 37. S30 OR S31 OR S32 OR S33 OR S34 OR S35 OR S36 38. S25 AND S29 AND S37 |

Online Supplement B – Literature Search 2 algorithm

| **CINAHL & MEDLINE (EBSCO), EMBASE, Google Scholar, Search Strategy**  **(limit 2010-2021, english language only. Search conducted March 15^th^ - April 7^th^, 2021)** |
| --- |
| 1. MH “Decision Making, Computer Assisted” AND 2. TI or AB “Nurs*” 3. TI or AB ‘Acute’ 4. TI or AB ‘Handheld’ |
| **Handsearch of journal index listings “CIN: Computers, Informatics, Nursing”, 2010 – 2021** |
| Search criteria:   1. Acute nursing practice focus 2. Bedside digital technology focus 3. Clinical decision-making focus |
| **Handsearch of the references lists of included articles all results** |
| Search criteria:   1. Acute nursing practice focus 2. Bedside digital technology focus 3. Clinical decision-making focus |

Online Supplement C – Literature Search 3 algorithm

| Line | Search Term |
| --- | --- |
| Population (staff / patients / setting) | |
| S1 | MH(“Nursing Staff, Hospital”) OR MH (“Inpatients”) |
| S2 | MH(“Acute Care Nurse Practitioner”) OR MH(“workflow”) |
| S3 | nurs* OR hospital* OR inpatien* OR (acute N3 care) |
| S4 | S1 OR S2 OR S3 |
| Intervention (stand-alone terms – includes concepts of computer PLUS decision making) | |
| S5 | MH(“Decision Support Systems, Clinical”) OR MH(Computer-Assisted Decision Making) OR MH(“Therapy Computer-Assisted”) OR MH(“Drug Therapy, Computer-Assisted”) |
| S6 | MH(“Decision Making, Computer Assisted”) OR MH(“Nursing Care Plans, Computeri?ed”) |
| S7 | computer* N2 (decision* OR “care plan” OR toolkit) |
| S8 | electronic* N2 (decision* OR “care plan” OR toolkit) OR “ehealth system*” |
| S9 | digital* N2 (decision* OR “care plan” OR toolkit) OR “clinical decision support” |
| S10 | S5 OR S6 OR S8 OR S8 OR S9 |
| Intervention (combined terms) | |
| S11 | MH(“Expert Systems”) OR MH(“Point-of-Care Systems”) OR MH(“Mobile devices”) OR MH(“Tablet Computers”) OR MH(“Smart Phone”) |
| S12 | MH(“Computers, Hand-Held”) OR MH(“Computers, Portable”) OR MH(“Computers and Computerization”) OR MH(“Mobile Applications”) OR MH(“Cloud Computing”) |
| S13 | “handheld computer*” OR “handheld device*” OR “handheld decision support” OR iphone OR i-phone OR smartphone OR smart?phone OR “cell* phone” OR ipad OR i-pad OR “tablet device*” OR “tablet computer*” OR “tablet PC*” OR “tablet technolog*” OR mhealth OR m-health OR “mobile health” OR mobile N1 (“information system*”) OR “touch?screen device*” OR (mobile N2 app*) OR (tablet N2 app*) OR “patient?facing applicatio*” OR “point?of?care” OR “bedside monitor” OR “information system*” OR “smart device*” OR “handheld computer*” OR (android N2 tablet) OR “hands free communication” OR “electronic nursing record” OR “real-time data” OR “technology intervention” OR “information technology” OR “electronic health records” OR “information system” |
| S14 | S11 OR S12 OR S13 |
| S15 | (MH(“Decision Making”) OR MH(“Decision Support Techniques”) OR MH(“Clinical Decision-Making)) |
| S16 | MH(“Decision Making, Clinical”) OR MH(“Decision Making, Patient”) OR MH (“Nursing Care Plans”) OR MH(“Patient Bedside”) OR MH(“Program Implementation”) OR MH(“Patient safety”) OR MH(“Teamwork”) |
| S17 | “clinical judgment*” OR “patient safety” OR “response system” OR “communication strategy” OR “infection prevention” OR “patient care” OR “nursing W0 (task* OR practice*) OR ((decision* N3 (care or make or makes or making or made or support* or algorithm* or aid or aids or app or apps or application* or technique*)) or expert system*) OR (nurs* N0 satisfaction) |
| S18 | S15 OR S16 OR S17 |
| Final combination | |
| S19 | S14 AND S18 |
| S20 | S10 OR S19 |
| S21 | S4 AND S20 |

Online Supplement D – Results of Structured Quality Appraisal

Risk of bias in included studies.

| **Design**  **/study (year)** | **Item 1** | **Item 2** | **Item 3** | **Item 4** | **Item 5** | **Item 6** | **Item 7** | **Item 8** | **Item 9** | **Item 10** |
| --- | --- | --- | --- | --- | --- | --- | --- | --- | --- | --- |
| **Randomised controlled trials** | **True randomization to assign pts. to treatment groups?** | **Allocation to treatment groups concealed?** | **Treatment groups similar at the baseline?** | **Participants blind to treatment assignment?** | **Those delivering treatment blind to treatment assignment?** | **Outcomes assessors blind to treatment assignment?** | **Treatment groups treated identically other than intervention of interest?** | **Follow up complete or described and analyzed?** | **Participants analyzed in the groups to which they were randomized?** | **Outcomes measured in the same way for treatment groups?** |
| Bakken et al., (2014) ^28^ | **+** | **?** | **-** | **-** | **-** | **?** | **+** | **+** | **+** | **+** |
|  | **Outcomes measured in a reliable way?** | **Appropriate statistical analysis used?** | **Trial design appropriate, and deviations from standard RCT design accounted for?** |  |  |  |  |  |  |  |
|  | **+** | **+** | **+** |  |  |  |  |  |  |  |
| Siebert et al., (2017)^29^ | + | + | + | - | - | - | + | + | + | + |
|  | + | + | + |  |  |  |  |  |  |  |
| Siebert et al., (2019)^30^ | + | + | + | - | - | - | + | + | + | + |
|  | + | + | + |  |  |  |  |  |  |  |
| **Quasi-experimental studies** | **‘Cause’ and ‘effect’ clear?** | **Pts included in comparisons similar?** | **Pts compared similar except for exposure/ intervention** | **Was there a control group?** | **Multiple outcomes pre and post intervention?** | **Was follow up complete?** | **Outcomes measured in the same way?** | **Outcomes measured in a reliable way?** | **Appropriate statistical analysis?** | **-** |
| Cleaver (2021)^31^ | **+** | **?** | **+** | **+** | **N/A** | **+** | **+** | **+** | **+** |  |
| Doran et al. 2010^39^ | **+** | **+** | **+** | **N/A** | **+** | **-** | **+** | **+** | **+** |  |
| Godwin (2015)^32^ | **+** | **+** | **+** | **+** | **+** | **?** | **+** | **+** | **+** |  |
| Johanssen 2012^34^ | **+** | **+** | **-** | **-** | **+** | **?** | **-** | **?** | **-** |  |
| Ruland^35^ | **+** | **+** | **?** | **+** | **+** | **+** | **?** | **+** | **+** |  |
| Sedgwick 2017^37^ | **+** | **+** | **+** | **-** | **+** | **?** | **+** | **+** | **+** |  |
| Sedgwick 2019^38^ | **+** | **+** | **-** | **-** | **+** | **?** | **?** | **?** | **+** |  |
| Sefton^33^ | **+** | **+** | **?** | **-** | **+** | **?** | **+** | **+** | **+** |  |
| Singh (2017)^36^ | **+** | **+** | **+** | **N/A** | **+** | **-** | **+** | **?** | **?** |  |
| **Cohort studies** | **Two groups similar and recruited from the same population?** | **Exposures measured similarly to assign people to groups?** | **Exposure measured in a valid and reliable way?** | **Confounding factors identified?** | **Strategies to deal with confounding factors stated?** | **Groups/pts. free of the outcome at the start of the study?** | **Outcomes measured in a valid and reliable way?** | **Follow up time reported and sufficient to be long enough for outcomes to occur?** | **Follow up complete or reasons to loss to follow up described and explored?** | **Strategies to address incomplete follow up utilized?** |
| Cato, Hyun & Bakken (2014)^41^ | N/A | N/A | + | + | + | + | + | + | + | N/A |
|  | **Appropriate statistical analysis used** |  |  |  |  |  |  |  |  |  |
|  | + |  |  |  |  |  |  |  |  |  |
| Spat^40^ | N/A | N/A | **+** | N/A | N/A | **+** | **+** | **+** | **+** | N/A |
|  | **+** |  |  |  |  |  |  |  |  |  |
| **Longitudinal observational study** | **Two groups similar and recruited from the same population?** | **Exposures measured similarly to assign people to groups?** | **Exposure measured in a valid and reliable way?** | **Confounding factors identified?** | **Strategies to deal with confounding factors stated?** | **Groups/pts. free of the outcome at the start of the study?** | **Outcomes measured in a valid and reliable way?** | **Follow up time reported and sufficient to be long enough for outcomes to occur?** | **Follow up complete or reasons to loss to follow up described and explored?** | **Strategies to address incomplete follow up utilized?** |
| Kerns (2021)^42^ | **N/A** | **N/A** | **+** | **+** | **+** | **+** | **+** | **+** | **+** | **+** |
|  | **Appropriate statistical analysis used?** |  |  |  |  |  |  |  |  |  |
|  | **+** |  |  |  |  |  |  |  |  |  |
| **Cross-sectional studies** | **Inclusion criteria clearly defined?** | **Subjects/ settings described in detail?** | **Exposure measured in a valid and reliable way?** | **Objective, standard criteria to measure condition?** | **Confounding factors identified?** | **Strategies for confounding factors stated?** | **Outcomes measured in a valid and reliable way?** | **Appropriate statistical analysis used?** |  |  |
| Hsiao (2012)^43^ | **+** | **+** | **+** | **?** | **+** | **?** | **+** | **+** |  |  |
| Johansson (2014)^44^ | **+** | **+** | **+** | **+** | **?** | **?** | **+** | **+** |  |  |
| Lin (2014)^45^ | **+** | **?** | **+** | **+** | **?** | **?** | **+** | **+** |  |  |
| McCulloh (2018)^46^ | **+** | **+** | **+** | **-** | **+** | **+** | **+** | **+** |  |  |
| Moore & Jayewardene (2014)^48^ | **+** | **+** | **+** | **+** | **+** | **+** | **+** | **+** |  |  |
| Shen (2018)^47^ | **?** | **?** | **+** | **+** | **+** | **+** | **+** | **+** |  |  |
| Yuan et al. 2013^49^ | **+** | **+** | **+** | **+** | **+** | **+** | **+** | **+** |  |  |
| **Qualitative Research** | **Congruity between philosophical perspective and methodology?** | **Congruity between methodology and research objectives?** | **Congruity between methodology and data collection methods?** | **Congruity between methodology and data analysis?** | **Congruity between methodology and interpretation of results?** | **Statement locating the researcher culturally or theoretically?** | **Researcher influence addressed?** | **Pts and their voices adequately represented?** | **Research ethical according to current criteria?** | **Conclusions flow from data analysis / interpretation?** |
| Farrell^6^ | **?** | **?** | **?** | **?** | **+** | **-** | **?** | **+** | **+** | **+** |
| Momtahan^53^ | **+** | **+** | **+** | **+** | **+** | **-** | **-** | **+** | **+** | **+** |
| Reynolds^54^ | **+** | **+** | **+** | **+** | **+** | **-** | **?** | **+** | **+** | **+** |
| Ricks^50^ | **-** | **+** | **+** | **+** | **+** | **?** | **+** | **+** | **+** | **+** |
| **Diagnostic Accuracy** | **Consecutive or random sample of patients enrolled?** | **Case control study avoided?** | **Avoid inappropriate exclusions?** | **Index test results interpreted without prior knowledge of results?** | **Pre-specified threshold used?** | **Reference standard likely to correctly classify target condition?** | **Reference standard interpreted without knowledge of index test** | **Appropriate interval between index test and reference standard?** | **Did all patients receive the same reference standard?** | **Were all patients included in the analysis?** |
| Kartika et al., (2021)^52^ | **+** | **+** | **+** | **+** | **+** | **+** | **+** | **?** | **+** | **+** |
| O’Donnell^51^ | **+** | **?** | **?** | **+** | **+** | **+** | **+** | **?** | **+** | **+** |

*Note.* ^+^Yes; ^-^No; ^?^unclear; ^N/A^not applicable, single group study
